# Improvement of Cs_3_Cu_2_I_5_ Single-Crystal Growth Process by YCl_3_ Additives: Cu^+^ Oxidation Inhibition and Precursor Colloid Stabilization

**DOI:** 10.3390/molecules31081354

**Published:** 2026-04-20

**Authors:** Wang Zhou, Tianyun Du, Chunqian Xu, Xiuxun Han

**Affiliations:** 1 Institute of Optoelectronic Materials and Devices, School of Materials Science and Engineering, Jiangxi University of Science and Technology, Ganzhou 341000, China; 2 National Rare Earth Functional Materials Innovation Center, Guorui Kechuang Rare Earth Functional Materials (Ganzhou) Co., Ltd., Ganzhou 341100, China

**Keywords:** inverse temperature crystallization method, high-quality single crystals, colloidal stabilization, scintillators

## Abstract

Cs_3_Cu_2_I_5_ single crystals are regarded as promising next-generation scintillators due to their large Stokes shift and low self-absorption characteristics. However, the cost-effective solution growth method faces critical challenges: the instability of colloidal precursors in solutions and the severe oxidation of Cu^+^ during crystal growth. This study innovatively introduces yttrium chloride (YCl_3_) as a dual-functional additive to address both issues simultaneously. The hydrolysis of YCl_3_ creates a controlled acidic environment, effectively suppressing the oxidation of Cu^+^; meanwhile, it enhances the stability of colloidal precursors by significantly increasing their surface charge and narrowing the particle size distribution. These synergistic effects enable the rapid growth (approximately 100 h) of near-centimeter-sized Cs_3_Cu_2_I_5_ single crystals with high crystallinity, without the need for inert gas protection. The optimized crystals exhibit exceptional performance: a photoluminescence quantum yield (PLQY) of 93.22% ± 0.47%, a scintillation decay time of 210.04 ns, and a light yield of ~738.14 pe/MeV. This YCl_3_-mediated growth strategy establishes an efficient approach for the solution-based synthesis of high-quality Cs_3_Cu_2_I_5_ single crystals, holding great significance for advancing high-sensitivity, environment-stable radiation detection applications such as medical diagnostics and nuclear safety monitoring.

## 1. Introduction

In recent years, radiation detectors have become increasingly vital across a wide range of applications, including medical diagnostics, customs inspection, and industrial non-destructive testing [1,2,3]. The performance of such detectors is fundamentally governed by the scintillator materials employed, which require meticulous optimization of key scintillation properties. An ideal scintillator necessitates the concurrent achievement of high light yield and fast decay kinetics [4,5,6]. Among emerging scintillator materials, the zero-dimensional perovskite single crystal Cs_3_Cu_2_I_5_ has attracted significant attention as a promising self-activated scintillator. It features self-trapped exciton (STE) emission accompanied by a large Stokes shift, which effectively suppresses self-absorption, along with a remarkable photoluminescence quantum yield (PLQY) [7,8]. With a high density of 4.51 g/cm^3^, arising from the presence of heavy Cs atoms, Cs_3_Cu_2_I_5_ also possesses superior radiation interaction cross-sections and stopping power. Its impressive high light yield surpasses that of conventional scintillators such as NaI:Tl and LYSO:Ce, while maintaining short decay lifetimes [9,10,11].

Current synthesis methods for Cs_3_Cu_2_I_5_ single crystals include Bridgman growth, vapor-assisted antisolvent processing, and inverse temperature crystallization (ITC) [12,13,14,15,16]. Each technique, however, presents certain limitations. The vapor-assisted antisolvent approach often suffers from uncontrolled nucleation, restricting crystal dimensions. Among these methods, inverse temperature crystallization (ITC) stands out as a promising route for mass production of high-performance Cs_3_Cu_2_I_5_ crystals. Nevertheless, existing ITC processes require extended growth periods (e.g., ~30 days) to obtain large, defect-free single crystals.

To further enhance scintillation performance, defect engineering has emerged as a promising strategy in the development of advanced scintillators. Approaches such as anion mixing and aliovalent doping/codoping have been widely employed to regulate defect states and carrier recombination processes. For instance, Mg^2+^ codoping in Lu_3_Al_5_O_12_:Ce has been demonstrated to suppress trapping-related slow components while significantly enhancing the fast scintillation components [17]. In contrast to these lattice-modification strategies, additive-assisted regulation of precursor chemistry and crystal growth provides an alternative pathway to control defect formation and crystal quality.

Fundamental investigations into metal halide perovskite crystallization mechanisms indicate that a dynamic equilibrium involving solvated ions, colloidal clusters, and coordination complexes exists in precursor solutions [14,18]. For instance, Flatken et al. quantitatively analyzed colloidal precursors in MAPbI_3_ via small-angle scattering (SAS), identifying iodoplumbate complexes as the dominant colloidal species [19]. Temperature-dependent colloidal dissolution-reconstruction processes govern the crystal growth, where heating promotes dissolution into solvated ions and cooling facilitates their reassembly. Given the similar inverse temperature solubility behavior of Cs_3_Cu_2_I_5_ in polar aprotic solvents such as DMF and DMSO, it is reasonable to infer that similar dissolution–reconstruction processes occur in its precursor solutions. Compared to solvated ions, colloidal precursors can store a higher density of ions within a confined solution volume, maintaining a sustainable supply for long-term crystal growth. However, such colloids are often unstable and may deposit directly onto growing crystal surfaces, introducing defects. Moreover, when crystallization is conducted under ambient conditions without inert gas protection, oxidation of Cu^+^ ions is inevitable, deteriorating scintillation performance through the following reaction [14]:

(1) $$4{\mathrm{Cu}}^{+}\;+{\;O}_{2}\;+\;4{\mathrm{OH}}^{-}\to \;4\mathrm{CuO}\;+\;2H_{2}O$$

These defects collectively impair optical transparency and degrade luminescence efficiency.

In this work, we develop an additive-assisted optimization strategy to overcome these challenges. By introducing yttrium chloride (YCl_3_) into the precursor solution prior to crystallization, we achieved rapid growth (~100 h) of high-quality Cs_3_Cu_2_I_5_ single crystals with dimensions up to 4 mm × 8 mm and exceptional phase purity. Inductively coupled plasma (ICP) analysis confirmed the absence of incorporated yttrium in the crystals. Simultaneously, YCl_3_ effectively suppresses Cu^+^ oxidation, as verified by X-ray photoelectron spectroscopy (XPS). The optimized crystals exhibit a high light yield of 738.14 pe/MeV, significantly surpassing the performance of crystals grown without YCl_3_ addition (545.85 pe/MeV). The PLQY of optimized crystals also exceeds a record-high value of 93.22 ± 0.47%.

## 2. Results and Discussions

**Figure 1:**
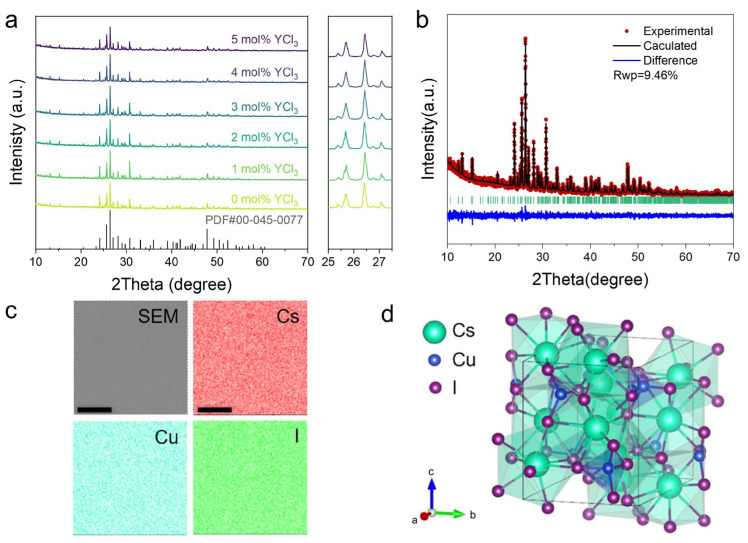
(**a**) XRD patterns of Cs_3_Cu_2_I_5_ single crystal powders added with varying concentrations of YCl_3_. (**b**) Rietveld refinement of Cs_3_Cu_2_I_5_ single crystal powder XRD data grown from a precursor solution with 2 mol% YCl_3_. (**c**) SEM image and EDS-mapping of a single crystal grown from a precursor solution with 2 mol% YCl_3_. Scale bar: 10 μm. (**d**) The calculated crystal structure of Cs_3_Cu_2_I_5_.

Pre-synthesized Cs_3_Cu_2_I_5_ seed crystals were introduced into the precursor solutions for single crystal growth via inverse temperature crystallization. Figure 1a presents the powder X-ray diffraction (XRD) patterns of Cs_3_Cu_2_I_5_ crystals synthesized with varying YCl_3_ concentrations (0–5 mol%). All diffraction peaks align well with the standard Cs_3_Cu_2_I_5_ reference (PDF#00-045-0077), and no other phase can be detected. The crystal growth process effectively excludes Y^3+^ incorporation, as confirmed by the ICP-MS results showing yttrium content at the parts-per-billion level (Table S1). Rietveld refinement of the XRD pattern for the 2 mol% YCl_3_-modified sample (Figure 1b) confirms the orthorhombic crystal structure (space group *Pnma*), with the lattice parameters detailed in Table S2. Energy-dispersive X-ray spectroscopy (EDS) mapping at 8000× magnification (Figure 1c) reveals a homogeneous distribution of Cs, Cu, and I in the resulting single crystals. The characteristic zero-dimensional host–guest structure (Figure 1d) consists of an isolated [Cu_2_I_5_]^3−^ polyhedron featuring two distinct copper coordination geometries—tetrahedral (Cu_1_) and trigonal planar (Cu_2_)—spatially separated by Cs^+^ cations. This structural arrangement confines electronic states within discrete polyhedrons, giving rise to zero-dimensional quantum confinement effects.

As previously reported, the solvents DMF and DMSO are hygroscopic [20,21,22,23]. The introduction of YCl_3_ into the DMF/DMSO solvent system can initiate hydrolysis under trace moisture conditions. The released H^+^ helps reduce dissolved oxygen levels via iodide oxidation according to the following reaction [24]:

(2) $$4I^{-}+{\;O}_{2}\;+\;4H^{+}\;\to \;2I_{2}\;+\;2H_{2}O$$

Subsequently, the I_2_ reacts with I^−^ to form I_3_^−^ via [25]:

(3) $$I^{-}+I_{2}\;↔\;I_{3}^{-}$$

**Figure 2:**
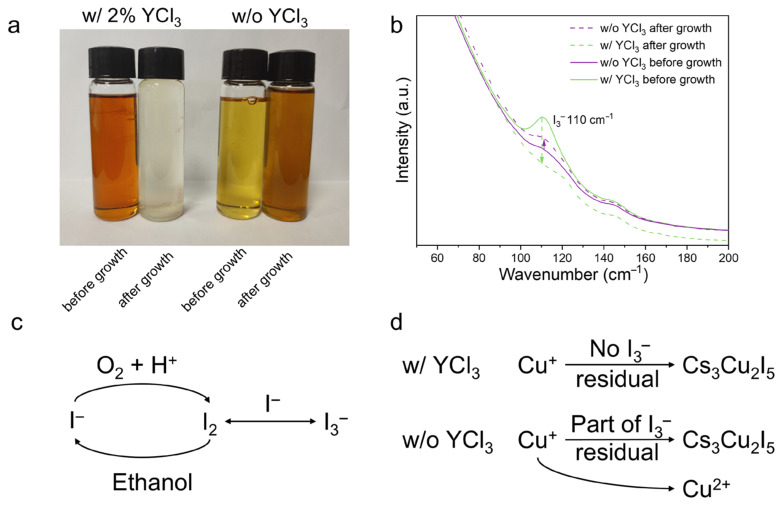
(**a**) Image of precursor solutions before (left) and after (right) crystallization. (**b**) Raman spectra of precursor solutions shown in (**a**). Transformation process of (**c**) iodine and (**d**) copper ions in precursor solution.

As shown in Figure 2a, the precursor solution containing 2 mol% YCl_3_ exhibits intense brown coloration compared to the YCl_3_-free solution, which only exhibits light yellow color. Raman spectroscopy (Figure 2b) further confirms this behavior, showing a pronounced I_3_^−^ vibrational peak near 110 cm^−1^ in YCl_3_-modified solutions before crystallization [26,27]. It can be inferred that the H^+^-facilitated I^−^ oxidation process establishes an oxygen-depleted environment in the precursor solution, which effectively suppresses the oxidative conversion of Cu^+^ to Cu^2+^. Furthermore, I_3_^−^ can be reduced back to I^−^ under heating conditions (consistent with the ITC process). At elevated temperatures during crystal growth, ethanol acts as a reducing agent to facilitate the reduction of I_2_ to I^−^ (shown in Figure 2c) [28]. These processes enable the cyclic recycling of iodine and replenish the consumption of I^−^ by crystal growth. In the precursor solution with added YCl_3_, the Cu^+^ and I^−^ in the precursor solution are gradually consumed in a stoichiometric molar ratio of Cs_3_Cu_2_I_5_ as crystallization proceeds. To maintain the stoichiometric balance required for continuous crystal growth, the pre-formed I_3_^−^ undergoes dissociation to release free I^−^, thereby replenishing the iodide ion pool in the system. Notably, the brown color of the precursor solution with 2 mol% YCl_3_ completely fades after crystal growth, which provides direct evidence for the full utilization of solute I^−^ (consistent with the Raman results in Figure 2b).

In contrast, the oxidation of I^−^ cannot proceed sufficiently at the initial stage of crystal growth in the precursor solution without YCl_3_, which is attributed to the lack of H^+^ generated by YCl_3_ hydrolysis, as verified by the relatively light color and low I_3_^−^ yield for the solution before growth (Figure 2a,b). This insufficiency implies that the residual dissolved oxygen in the solution is likely to induce severe oxidative conversion of Cu^+^ to Cu^2+^, failing to effectively inhibit Cu^+^ loss. During crystal growth, the oxidative loss of Cu^+^ leads to a deviation from the 2:5 Cu^+^/I^−^ stoichiometric ratio and allows part of the I^−^ to remain in the precursor solution in the form of I_3_^−^. Consequently, the precursor solution without YCl_3_ retains a distinct brown tint even after crystal growth, as shown in Figure 2a, and the Raman spectrum shows an increased I_3_^−^ vibrational peak at ~110 cm^−1^ in Figure 2b. These insufficiently reduced I_3_^−^ species and the oxidized Cu^2+^ can be readily incorporated into the Cs_3_Cu_2_I_5_ single crystals, leading to a deterioration of the scintillation performance.

**Figure 3:**
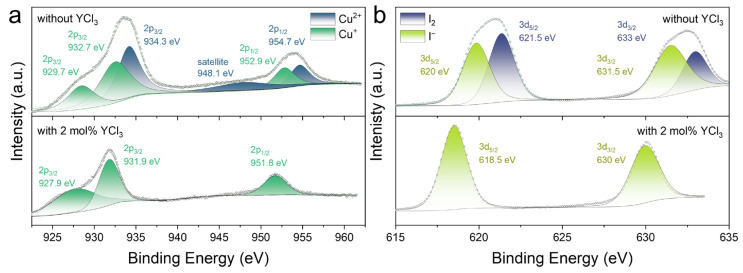
High-resolution XPS spectra of Cu 2p (**a**) and I 3d (**b**) orbitals of Cs_3_Cu_2_I_5_ single crystals.

To further validate the antioxidative role of YCl_3_ and confirm whether Cu^2+^ is incorporated into the Cs_3_Cu_2_I_5_ single crystals, we employed X-ray photoelectron spectroscopy (XPS) to analyze the copper valence states in the Cs_3_Cu_2_I_5_ single crystals. As presented in the EDS spectrum and full XPS spectra (Figures S1 and S2), only characteristic signals of Cs, Cu, and I were detected, with no Y-related binding energy peaks being observed, confirming the elemental purity of the crystals and the exclusion of Y^3+^ during growth. For the Cu 2p region (Figure 3a), peaks corresponding to Cu^2+^ were identified at 934.3 eV (2p_3/2_), 948.1 eV (Cu^2+^ shake-up satellite) and 954.7 eV (2p_1/2_) in crystals grown without YCl_3_, clearly indicating significant oxidation of Cu^+^ [29,30]. In contrast, YCl_3_-modified crystals predominantly exhibited Cu^+^-associated peaks: 927.9 eV (trigonal-coordinated Cu^+^ 2p_3/2_), 931.9 eV (tetrahedral-coordinated Cu^+^ 2p_3/2_), and 951.8 eV (Cu^+^ 2p_1/2_), indicating that the oxidation of Cu+ has been efficiently suppressed.

The I 3d XPS fine spectra (Figure 3b) further support the regulatory role of YCl_3_ in the crystal growth process. These single crystals exhibit two binding energy peaks at about 620 eV and 632 eV, which are attributed to I 3d_5/2_ and I 3d_3/2_ [31,32,33]. The binding energy peaks of single crystals without the addition of YCl_3_ consist of two components, corresponding to I^−^ and I_2_ [34]. Conversely, in YCl_3_-modified crystals, only I^−^-specific binding energy peaks were detected at ~618.5 eV (3d_5/2_) and ~630.0 eV (3d_3/2_), demonstrating complete retention of iodide in its reduced form. As inferred from earlier analysis, the formation of I_2_ is driven by the excessive consumption of Cu^+^ (via oxidation to Cu^2+^), which disrupts the balanced Cu^+^/I^−^ ratio in the precursor. As the temperature rises, the solubility of colloidal precursors decreases, leading to their direct deposition onto crystal surfaces without sufficient solvation and causing insufficient solvation of iodine. These Cu^2+^ and I_2_ species have an adverse effect on the luminescence and scintillation performance of Cs_3_Cu_2_I_5_ single crystals.

Beyond suppressing the oxidative conversion of Cu^+^ to Cu^2+^ (evidenced by XPS spectra in Figure 3a), YCl_3_ also plays a critical role in regulating the stability of colloidal precursors in the solution—a key factor that directly governs the crystallinity and defect density of the resulting single crystals. The aggregation and dispersion behavior of colloidal precursors is fundamentally dictated by the competitive balance between two interparticle forces: repulsive Coulombic interactions (originating from surface charges) and attractive van der Waals forces [35,36].

**Figure 4:**
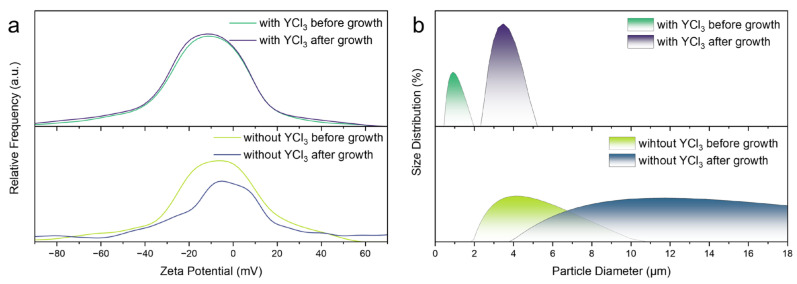
ζ-potential curves (**a**) and particle distribution curves (**b**) of precursor solution with and without YCl_3_, obtained before growth and after growth.

Upon introducing YCl_3_ into the DMF/DMSO-based precursor system, the H^+^ generated via YCl_3_ hydrolysis facilitates the controlled dissolution of colloidal precursors. This acid-enhanced dissolution process not only reduces the size of colloidal clusters but also increases their specific surface area, which in turn elevates the surface charge density of the colloids. To quantify this effect, ζ-potential measurements were conducted on precursor solutions (with and without 2 mol% YCl_3_) before and after the 100 h ITC process. As shown in Figure 4a, the absolute ζ-potential of colloidal precursors before growth increases from 7.0 to 11.6 mV upon YCl_3_ introduction, with only a minimal decrease to 10.8 mV after crystal growth. This enhanced electrostatic repulsion effectively counteracts attractive van der Waals forces, thereby sustaining the stable dispersion of colloidal precursors throughout the entire crystal growth period. In stark contrast, the YCl_3_-free precursor solution exhibits a sharp drop in the absolute ζ-potential to 5 mV after growth, indicating severe colloidal aggregation that disrupts the steady supply of solvated ions for crystal growth.

Dynamic light scattering (DLS) analysis further confirms the stabilizing effect of YCl_3_ on colloidal precursors (Figure 4b). The YCl_3_-free solution displays a broad particle size distribution (PSD), ranging from 2 to 10 μm. This broad distribution is attributed to uncontrolled aggregation driven by dominant van der Waals forces during heating. In contrast, the 2 mol% YCl_3_-modified solution maintains monodisperse colloidal precursors with a narrow PSD of 0.5–2 μm. Even after 100 h of ITC growth, the PSD of the YCl_3_-modified system only expands slightly to 2–5 μm, whereas the YCl_3_-free system exhibits further aggregation and an expanded PSD. This well-constrained particle size evolution is directly correlated with the sustained ζ-potential stability induced by YCl_3_, as the elevated surface charge density prevents excessive colloidal clustering. Collectively, the dual role of YCl_3_ (suppressing Cu^+^ oxidation and stabilizing colloidal precursors) lays the foundation for the rapid growth of high-quality Cs_3_Cu_2_I_5_ single crystals.

**Figure 5:**
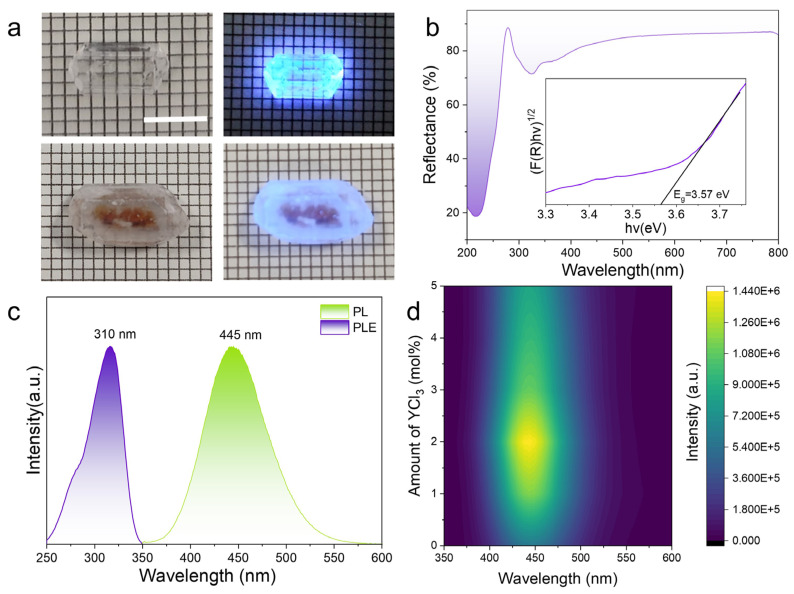
(**a**) Images of single crystals grown from solutions with 2 mol% YCl_3_ (top) and without YCl_3_ (bottom), along with their luminescence under 310 nm light. Scale bar: 5 mm. DRS (**b**), PL and PLE (**c**) of single crystal grown from the solution with 2 mol% YCl_3_. (**d**) PL spectra of single crystals grown from solutions with varying concentrations of YCl_3_.

Figure 5a presents the Cs_3_Cu_2_I_5_ single crystals grown with and without YCl_3_ addition. Although both systems enable large-scale crystal growth within 100 h, the crystals synthesized with YCl_3_ exhibit notably superior optical transparency and well-defined crystallographic facets. Figure 5b illustrates the diffuse reflectance spectroscopy (DRS) profile of the single crystal grown from the precursor solution containing 2 mol% YCl_3_. To determine the optical band gap (E_g_) of this crystal, the Kubelka–Munk function was employed, following the equations below [37]:

(4) $${\left(F\left(R\right)hv\right)}^{\frac{1}{2}}\;=\;A\left(hv\;-\;E_{g}\right)$$

(5) $$F\left(R\right)=\frac{{\left(1\;-\;R\right)}^{2}}{2R}$$

where R denotes the reflectance (in %), F(R) is the Kubelka–Munk function, hv represents the photon energy, and A is an optical constant. The E_g_ is calculated to be 3.57 eV, consistent with previously reported band gap data for Cs_3_Cu_2_I_5_ copper halide perovskites [38,39].

Under 310 nm excitation, the YCl_3_-optimized crystals emit intense STE luminescence centered at 445 nm, corresponding to a large Stokes shift of ~133 nm. This significant Stokes shift is critical for suppressing self-absorption, a key advantage of Cs_3_Cu_2_I_5_ in scintillation applications [40,41]. As shown in the PL spectra (Figure 5d), the PL intensity of Cs_3_Cu_2_I_5_ crystals exhibits a strong dependence on YCl_3_ concentration: compared to the YCl_3_-free control, the PL intensity of crystals grown with 2 mol% YCl_3_ is enhanced by 2.3-fold, and the corresponding photoluminescence quantum yield (PLQY) reaches 93.22% ± 0.47% (Figure S3). In addition, the YCl_3_-optimized crystals also exhibit good stability under ambient atmospheric conditions, maintaining 78.9% of the initial luminescence intensity after 60 days (Figure S4). Notably, excessive YCl_3_ addition (>2 mol%) leads to a distinct decrease in PL intensity. This phenomenon can be attributed to the overproduction of H^+^ from YCl_3_ hydrolysis. As supported by ζ-potential (Figure 4a) and DLS (Figure 4b) analyses, excessive H^+^ causes over-fragmentation and dispersion of precursor colloids, which promotes the formation of an excessively high concentration of solvated ions. This uncontrolled increase in solvated ion concentration accelerates the crystallization rate beyond the equilibrium range, leading to the formation of subtle lattice defects that act as non-radiative recombination centers, ultimately reducing the PL intensity.

**Figure 6:**
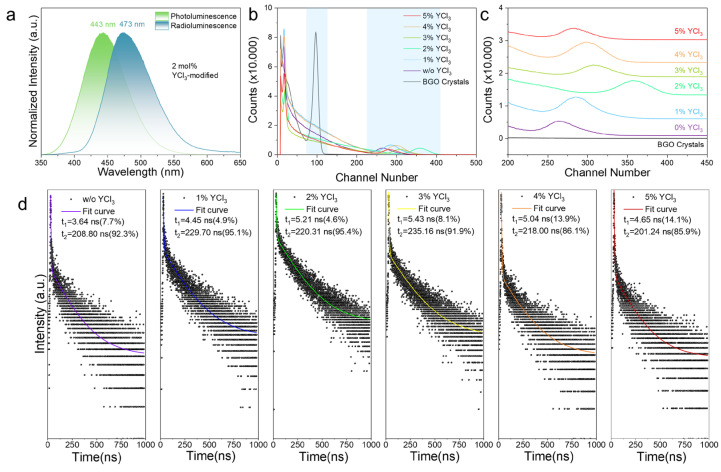
(**a**) Normalized PL and RL spectra of single crystals grown from solution with 2 mol% YCl_3_. (**b**) Pulse height spectra of Cs_3_Cu_2_I_5_ and BGO single crystals grown from precursor solutions with gradient concentrations of YCl_3_, measured under ^137^Cs radiation. (**c**) A magnified view of (**b**). (**d**) Scintillation decay curve of Cs_3_Cu_2_I_5_ single crystals grown from solutions with varying concentrations of YCl_3_.

RL spectra of the YCl_3_-modified Cs_3_Cu_2_I_5_ samples were measured under X-ray irradiation (tungsten target, 40 kV), as presented in Figure 6a. The RL spectrum of the optimized sample exhibits a broadband emission (410–600 nm) centered at 483 nm, with a 38 nm redshift relative to the PL spectrum, consistent with previous reports [42,43]. This spectral shift originates from X-ray-induced population of low-energy triplet states that dominate STE recombination pathways under high-energy excitation. According to the dipole selection rule, the 310 nm excitation favors spin-allowed transitions from the ground state to singlet states, while X-ray excitation can efficiently populate triplet states via ionization processes. Since the emission of Cs_3_Cu_2_I_5_ involves both higher-energy singlet-related STE emission and lower-energy triplet-related STE emission, the enhanced contribution of the lower-energy triplet channel under X-ray excitation shifts the emission to longer wavelengths compared with the PL spectrum [44]. The scintillation decay kinetics of the samples (Figure 6b) conform to a biexponential decay model, described by the following equation [26]:

(6) $$I\left(t\right)={\;I}_{0}+{\;A}_{1}\exp\left(\frac{-t}{\tau_{1}}\right)+A_{2}\exp\left(\frac{-t}{\tau_{2}}\right)$$

where I(t) denotes the time-dependent RL intensity, I_0_ is the initial intensity at t = 0, A_1_ and A_2_ are the amplitude constants corresponding to the two decay components, and τ_1_ (fast component) and τ_2_ (slow component) are the respective decay time constants. The average decay time is calculated by:

(7) $$\tau_{\mathrm{ave}}\;=\;\frac{A_{1}\tau_{1}^{2}\;+{\;A}_{2}\tau_{2}^{2}}{A_{1}\tau_{1}\;+{\;A}_{2}\tau_{2}}$$

As the YCl_3_ concentration increases, the average scintillation decay time of the single crystals first increases and then decreases. The average decay time is strongly correlated with the defect density in the crystals. As discussed earlier, Cs_3_Cu_2_I_5_ single crystals grown without YCl_3_ exhibit relatively high defect density, primarily attributed to the formation of Cu^2+^ (from Cu^+^ oxidation) and I_3_^−^ (from excessive I^−^ oxidation). In contrast, the addition of excessive YCl_3_ (>2 mol%) leads to uncontrolled supersaturation in the precursor solution, which accelerates the crystallization rate beyond the equilibrium range and disrupts the stability of colloidal precursors, ultimately introducing new defects. At the optimal YCl_3_ concentration (2 mol%), the crystal achieves the lowest defect density and an average scintillation lifetime τ_ave_ of 210.04 ns. To quantify the scintillation light yield, the gamma-ray pulse height spectroscopy was performed using a ^137^Cs radiation source, as shown in Figure 6c. Calibration was conducted with reference to the known light yield of commercial BGO scintillators (200 pe/MeV), using the channel number of the full-energy peak as the quantitative metric. The results show a concentration-dependent enhancement in light yield, with a maximum of 738.14 pe/MeV achieved at 2 mol% YCl_3_, representing a 30.3% improvement compared to Cs_3_Cu_2_I_5_ crystals without the addition of YCl_3_. This nonlinear optimization trend, where the light yield first increases then decreases with YCl_3_ concentration, stems from the two above-mentioned competing effects: YCl_3_-mediated defect suppression (YCl_3_ ≤ 2 mol%) versus colloidal destabilization-induced disorder (YCl_3_ > 2 mol%). Notably, the light yield of the optimized Cs_3_Cu_2_I_5_ crystal is 3.7 times higher than that of commercial BGO scintillators, highlighting their significant potential for application in high-sensitivity radiation detection systems.

## 3. Materials and Methods

### 3.1. Chemicals

All chemicals were used as received without purification: cesium iodide (CsI, 99.9%, Macklin, Shanghai, China), cuprous iodide (CuI, 99.9%, Macklin), N,N-Dimethylformamide (DMF, 99.8%, Aladdin, Riverside, CA, USA), dimethyl sulfoxide (DMSO, 99.8%, Aladdin), yttrium chloride hexahydrate (YCl_3_·6H_2_O, 99.9%, Macklin), anhydrous ethanol (AR, 99.7%, Sinopharm, Shanghai, China).

### 3.2. Synthesis of Cs_3_Cu_2_I_5_ Seed Crystal

The Cs_3_Cu_2_I_5_ seed crystals were synthesized via antisolvent vapor-assisted crystallization. A stoichiometric mixture of 7.79 g CsI and 3.808 g CuI was dissolved in 16 mL DMF and 4 mL DMSO. After 12 h of magnetic stirring, anhydrous ethanol was then incrementally added until the solution exhibited turbidity, followed by filtration through 0.45 μm PTFE membranes to obtain a clear yellow precursor. The solution was transferred to a 50 mL three-neck flask (A) connected via a gas-tight connection to another flask (B) containing ethanol. Flask A was maintained at 60 °C and flask B at 50 °C for 48 h, allowing controlled ethanol vapor diffusion from flask B to flask A. Upon completion of the reaction, the seed crystals were filtered and washed with ethanol and dried at 60 °C for 2 h.

### 3.3. Synthesis of Cs_3_Cu_2_I_5_ Single Crystal

For bulk crystal growth, the inverse temperature crystallization was employed. Typically, precursors of 11.685 g CsI, 5.712 g CuI, and variable concentrations (0–5 mol%) of YCl_3_·6H_2_O were dissolved in 24 mL DMF and 6 mL DMSO. The mixture was stirred at 60 °C for 12 h, and then anhydrous ethanol was added until the solution exhibited turbidity. After filtration, the reddish-brown solution was transferred to a 40 mL glass vial and one Cs_3_Cu_2_I_5_ seed crystal was added. The sealed vial was heated from 60 °C to 80 °C over 100 h (0.2 °C/h), followed by another 2 h of heating at 80 °C.

### 3.4. Characterizations

Structural characterization utilized a Tongda TD-3700 X-ray diffractometer (Cu-Kα, λ = 1.5406 Å) (Dandong, China) with 0.02° step resolution across 10–80°, while Rietveld refinement was employed via GSAS-II software (v5.7.4) [45]. Raman spectroscopy data were acquired using a Thermo Fisher (Waltham, MA, USA) DXR 2XI Raman spectrometer. Chemical states were analyzed using a ULVAC-PHI PHI 5000 VersaProbe III XPS system (Chigasaki, Japan). Colloidal behavior was quantified through ζ-potential measurements and DLS size distributions using an Anton Paar (Graz, Austria) Litesizer 500 dynamic light scattering instrument.

Optical properties (PL and PLE spectra) were obtained via a Horiba (Kyoto, Japan) Fluoromax+ fluorescence spectrophotometer, with an integrating sphere for PLQY measurements under xenon lamp excitation. The X-ray excited luminescence spectrum was characterized by a tungsten-source X-ray tube (40 kV, 20 mA) coupled to a Hamamatsu 7ID101-CR131 PMT detector (Hamamatsu, Japan). Gamma-ray responses were quantified using a 137Cs source with a Hamamatsu R2059 PMT and Ortec signal processing chain, calibrated against BGO standards (200 pe/MeV). The energy resolution of the single crystal was obtained using the half-width of the full-energy peak in the pulse height spectrum. Scintillation decay kinetics (210.04 ns) were resolved through time-correlated single photon counting (TCSPC) using dual Hamamatsu R1828-01 PMTs and Ortec CFD/TAC modules.

## 4. Conclusions

This study demonstrates that YCl_3_ acts as a dual-functional additive to optimize the growth of Cs_3_Cu_2_I_5_ single crystals. The hydrolysis of YCl_3_ generates a moderate amount of H^+^, which effectively suppresses the oxidation of Cu^+^. Meanwhile, YCl_3_ enhances the stability of colloidal precursors in the growth solution by increasing the absolute value of ζ-potential and narrowing the particle size distribution. These synergistic effects enable the rapid growth of high-quality Cs_3_Cu_2_I_5_ single crystals without the need for inert gas protection. The YCl_3_-optimized Cs_3_Cu_2_I_5_ single crystals exhibit significantly reduced defect density, improved optical transparency, and superior scintillation performance, with a photoluminescence quantum yield (PLQY) of 93.22% ± 0.47% and a light yield of approximately 738.14 pe/MeV. These performance metrics outperform commercial BGO scintillators and also demonstrate excellent long-term stability, highlighting the potential of this YCl_3_-mediated growth strategy for advancing Cs_3_Cu_2_I_5_ single crystals in high-sensitivity radiation detection applications.

## Data Availability

The original contributions presented in this study are included in the article and Supplementary Material. Further inquiries can be directed to the corresponding author.

## Supplementary Materials

The following supporting information can be downloaded at: https://www.mdpi.com/article/10.3390/molecules31081354/s1. Table S1: Concentration of Y in Cs_3_Cu_2_I_5_ single crystals with addition of varying concentration of YCl_3_, data acquired with ICP-MS. Table S2: Single-crystal crystallographic data of Cs_3_Cu_2_I_5_ single crystal grown from precursor solutions with 2 mol% YCl_3_. Figure S1: EDS spectrum of single crystals grown from a precursor solution with the addition of 2 mol% YCl_3_. Figure S2: XPS spectrum of single crystals grown from the precursor solution with 2 mol% YCl_3_ addition (a) and without YCl3 (b). Figure S3: The PLQY measurements of Cs_3_Cu_2_I_5_ single crystals grown from the precursor solution with 2 mol% YCl_3_ addition. The measurements were repeated for six times, and the PLQY of the sample is 93.22 ± 0.47%. Figure S4: Luminescence stability testing of Cs_3_Cu_2_I_5_ single crystals (modified by 2 mol% YCl_3_) under ambient atmospheric conditions.
