# Supplementary material for: Improvement of Cs3Cu2I5 Single-Crystal Growth Process by YCl3 Additives: Cu+ Oxidation Inhibition and Precursor Colloid Stabilization
Source: Molecules. 2026 Apr 20;31(8):1354. doi: 10.3390/molecules31081354 (PMC13119263; doi:10.3390/molecules31081354)
Supplement: Supplementary file 1 [file molecules-31-01354-s001.zip › molecules-4250154-supplementary.pdf]

Supporting Information

Oxidation-Suppressive Colloidal Stabilization Strategy Enables Rapid Growth of High-Performance $Cs_3Cu_2I_5$ Scintillator Crystals

Wang zhou[1], Tianyun Du[1], Xiuxun Han[1,2,*]

1 Institute of Optoelectronic Materials and Devices, School of Materials Science and Engineering, Jiangxi University of Science and Technology, Ganzhou, 341000, China

2 Guorui Kechuang Rare Earth Functional Materials (Ganzhou) Co., Ltd. (National Rare Earth Functional Materials Innovation Center), Ganzhou, 341100, China

*Corresponding author.

E-mail address: xxhan@jxust.edu.cn (Xiuxun Han)

Table S1 Concentration of Y in $Cs_3Cu_2I_5$ single crystals with addition of varying concentration of YCl3, data acquired with ICP-MS

Sample	Concentration of Y(ppb)
1 mol% YCl_3	21.6
2 mol% YCl_3	10.5
3 mol% YCl_3	26.9
4 mol% YCl_3	15.9
5 mol% YCl_3	20.4

Table S2 Single-crystal crystallographic data of Cs_3Cu2I_5 single crystal grown from precursor solutions with 2 mol% YCl_3

Empirical formula	$Cs_3Cu_2I_5$
Crystal system	orthorhombic
Space group	Pnma
Cell Length a (Å)	14.3637
Cell Length b (Å)	10.1764
Cell Length c (Å)	11.6544
α (°)	90
β (°)	90
γ (°)	90
Cell Volume ($Å^3$)	1703.53
χ^2	1.45
R_{wp}	9.46%

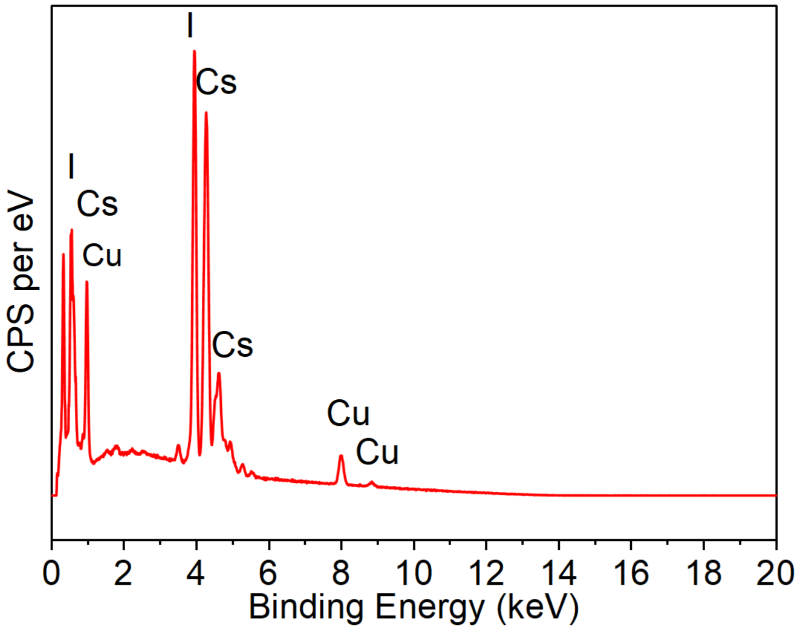

Figure S1. EDS spectrum of single crystals grown from a precursor solution with the addition of 2 mol% YCl_3

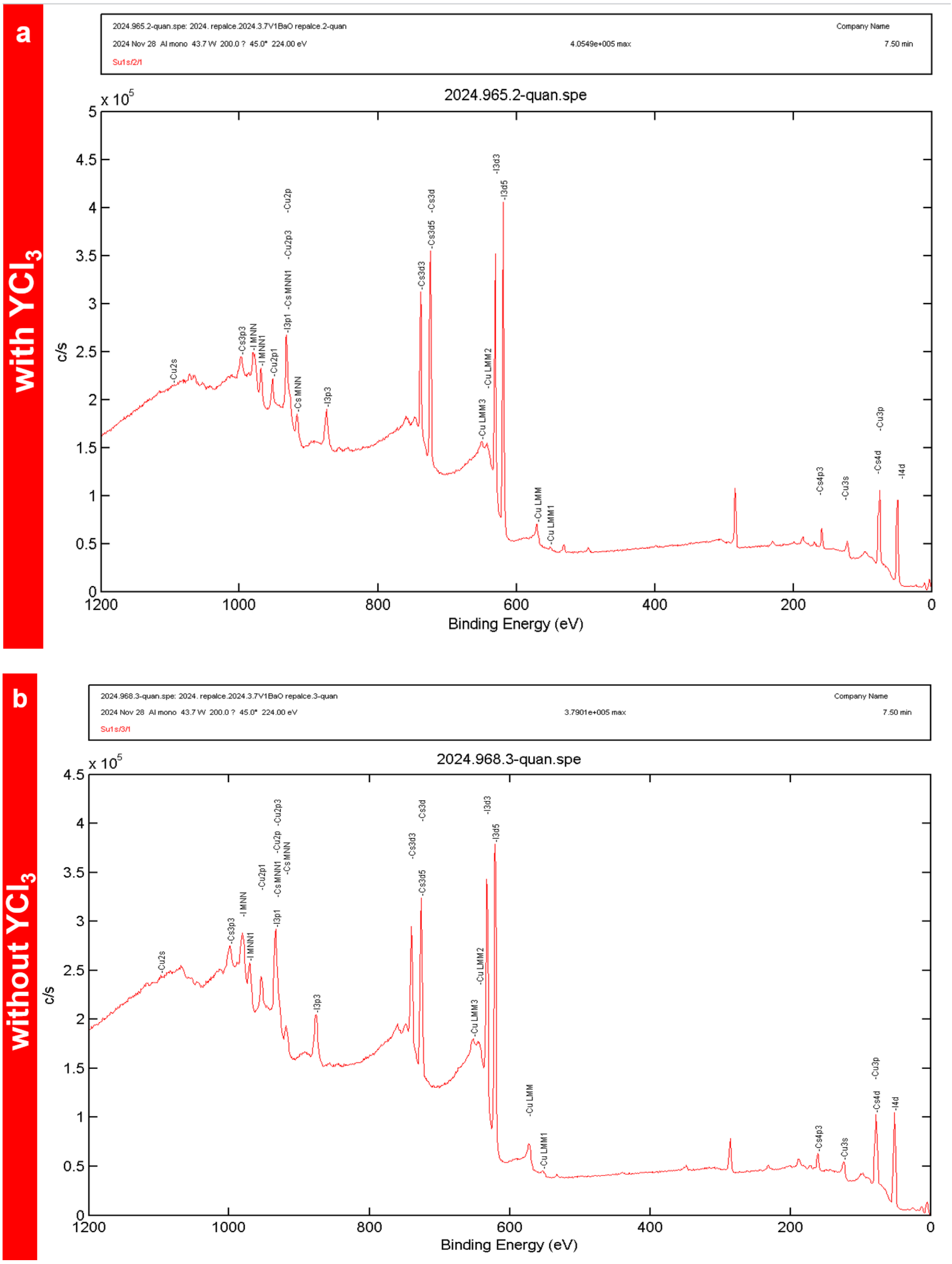

Figure S2. XPS spectrum of single crystals grown from the precursor solution with 2 mol% YCl_3 addition (a) and without YCl_3 (b).

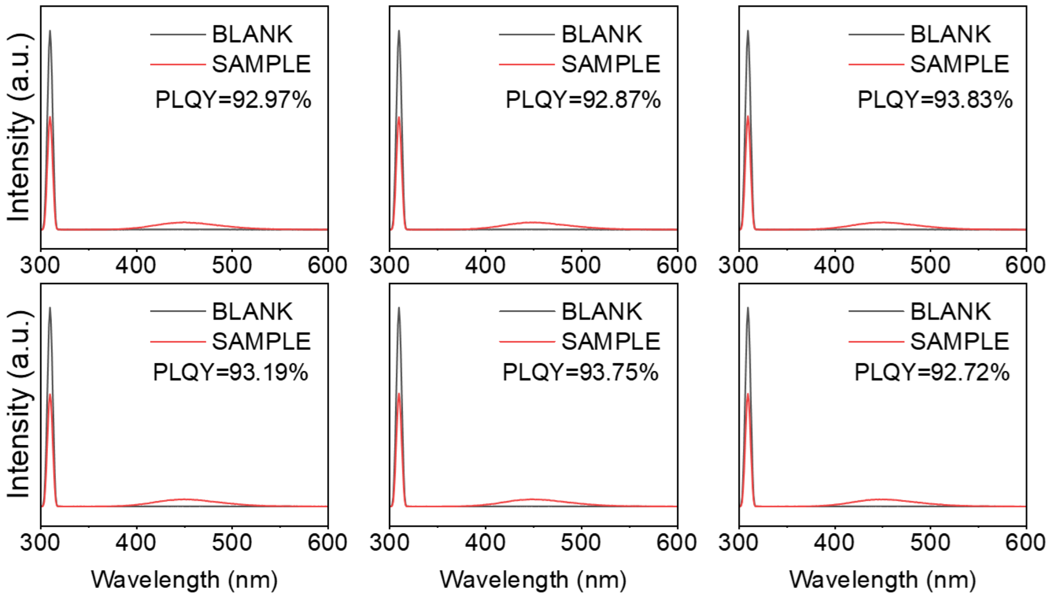

Figure S3. The PLQY measurements of $Cs_3Cu_2I_5$ single crystals grown from the precursor solution with 2 mol% YCl_3 addition. The measurements were repeated for six times, and the PLQY of the sample is 93.22 ± 0.47%.

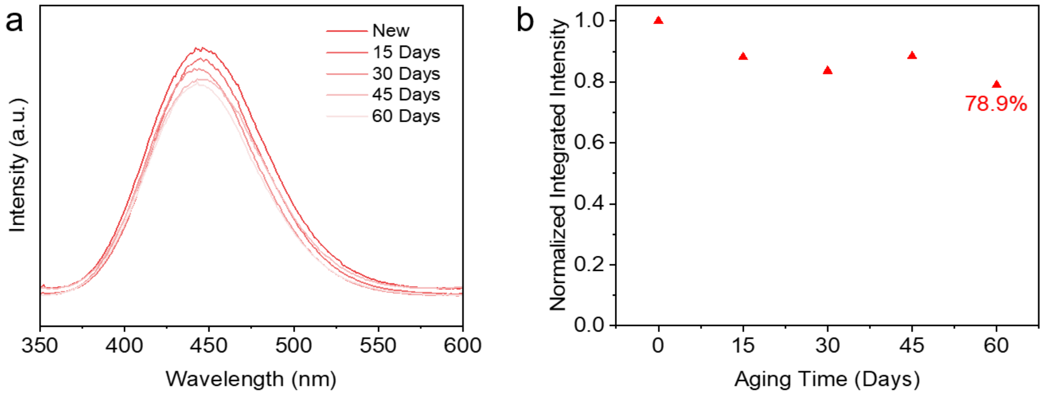

Figure S4. Luminescence stability testing of $Cs_3Cu_2I_5$ single crystals (modified by 2 mol% YCl_3) under ambient atmospheric conditions
